# FACTORS ASSOCIATED WITH CUSTODIAL GRANDCHILDREN RECEIVING MENTAL HEALTH CARE ASSISTANCE

**DOI:** 10.1093/geroni/igad104.2645

**Published:** 2023-12-21

**Authors:** Julian Montoro-Rodriguez, Bert Hayslip

**Affiliations:** University of North Carolina Charlotte, Charlotte, North Carolina, United States; University of North Texas, Denton, Texas, United States

## Abstract

The number of grandparents caring for grandchildren has increased substantially over the past two decades. Grandparent caregivers experience strain and family vulnerabilities as they face challenging child-care responsibilities, less social support, and more adverse emotional and lifestyle changes. These challenges may influence the type of support they provide to their grandchildren, affecting their grandchildren’s health and access to supportive services. In this light, research suggests that rates of service use among grandchildren are low (37% and 51% for community and school-based services) with the grandchild’s externalizing symptoms and medical/psychiatric diagnoses being the strongest predictors of such use. This paper investigates the influence of grandparent and grandchild-related factors associated with a grandchild’s receiving mental health care assistance. We examine how grandchildren’s use of counseling services affects grandparent well-being and relationship quality with the grandchild. Guided by the Pearlin stress-outcome paradigm (1990), we used data from 239 grandparent caregivers (Mage = 58.06, SD = 8.17), wherein 80 grandchildren were receiving counseling mental care and 159 were not. Predictors of well-being and quality of grandchild/grandparent relationship outcomes were regressed on the use of the counseling services, stressors (health of grandparent and problem behaviors of grandchildren), and coping resources (resiliency, self-efficacy, social support) using a SEM model with AMOS. Results indicate good model fit (CFI = 0.968; TLI= 0.959; RMSE = 0.04). Findings suggested that grandchildren’s use of mental health services influences both the quality of the relationship and the well-being of the grandparent albeit through different coping resources (grandparent’s resilience and social support).

